# PReS-FINAL-2252: Descriptive analysis of pediatric autoimmune neuropsychiatric disorder associated with streptococcus infection (PANDAS) in a cohort of 65 Italian patients

**DOI:** 10.1186/1546-0096-11-S2-P242

**Published:** 2013-12-05

**Authors:** F Falcini, G Lepri, D Rigante, F Bertini, M Matucci Cerinic

**Affiliations:** 1 Internal Medicine, Rheumatology Section, Transition Clinic, University of Florence, Florence, Italy; 2 Institute of Paediatrics, Università Cattolica Sacro Cuore, Rome, Italy; 3 Internal Medicine, Rheumatology Section, University of Florence, Florence, Italy

## Introduction

PANDAS includes neuropsychiatric symptoms, mainly obsessive-compulsive disorder (OCD) or tics, temporally associated with an immune-mediated response to group A β-hemolytic *Streptococcus *(GAS) infections, which suddenly start before puberty and display remitting/relapsing course.

## Objectives

To describe clinical features of a cohort of 65 Italian pts with previous confirmed GAS infection and sudden occurrence of OCD and/or tics.

## Methods

Descriptive analysis of a cohort of Italian patients with PANDAS.Between may 2009 - may 2013 we observed 65 pts (50 M, 15 F, mean age: 100.8 ± 32,9 months) with OCD and/or tics starting before puberty, associated with a previous GAS infection. Demographic and familiar data, routine and specific laboratory data: thyroid function, autoimmunity tests (ANA, anti-dsDNA, anti-ENA, anti-cardiolipin, and anti-tissue transglutaminase antibodies) were collected. 55/65 underwent brain MRI, all EEG, echocardiography and neuropsychiatric evaluation.

## Results

13/65 pts (20%) born from Caesarean section, 60/65 (92.3%) full-term, 32/65 (49,2%) had familiars with OCD/tics or other neurologic diseases, and 57/65 (87.7%) did sports. Acute and dramatic onset occurred at a mean age of 74.3 ± 25.9 months (range 24-160). Out of 65 pts, 22 (33.8%) started with motor tics; 2 (3.1%) with OCD; 5 (7.7%) with motor/vocal tics; 28 (43,1%) with motor tics and OCD; 1 (1.5%) with vocal tics and compulsive behavior and 7 (10.8%) with motor/vocal tics and OCD. 41/65 patients (63.1%) had previously pharyngitis, otitis and/or upper airway infections, 1 impetigo. Brain MRI, EEG, thyroid tests, and coeliac disease screening were normal in all. Mean age to diagnosis 101.2 ± 29.9 months. At onset and at our first clinical evaluation inflammatory parameters were negative in all. Five (7.7%) had a*nti-streptolysin *O titer less than 250 lU, 20 (30.8%) between 250 and 550 IU, 29 (44.6%) over 500 IU. Anti-DNase titer was increased (650-1200 U) in 38 (58,5%). All pts received benzathine benzylpenicillin. Sertraline or haloperidol were added in 10, and a psychotherapeutic help in 6. Seventy eight, 5% pts displayed complete or partial remission of initial symptoms.

## Conclusion

Our preliminary data show that PANDAS is mainly observed in males with familiar recurrence of different motor disturbances, clinical onset occurs at a mean age of 6 ± 2 years with the coexistence of OCD and motor tics in most cases, and increased anti-DNase titer is found in 58.5% of patients. Benzathine benzylpenicillin has resulted effective in 78.5% of cases with complete/partial remission of symptoms. New studies on large prospective cohorts of patients of different age and the identification of reliable biomarkers indicating early diagnosis and outcome of PANDAS are needed.

## Disclosure of interest

None declared.

